# Liquid bidentate ligand for full ligand coverage towards efficient near-infrared perovskite quantum dot LEDs

**DOI:** 10.1038/s41377-024-01704-x

**Published:** 2025-01-07

**Authors:** Zong-Shuo Liu, Ye Wang, Feng Zhao, Hua-Hui Li, Wei-Zhi Liu, Wan-Shan Shen, Hong-Wei Duan, Ya-Kun Wang, Liang-Sheng Liao

**Affiliations:** 1 Institute of Functional Nano & Soft Materials (FUNSOM), Jiangsu Key Laboratory for Carbon-Based Functional Materials & Devices, Soochow University, Suzhou, China; 2 Macao Institute of Materials Science and Engineering, Macau University of Science and Technology, Macau SAR, Taipa China

**Keywords:** Inorganic LEDs, Quantum dots

## Abstract

Perovskite quantum dots (PQDs) show promise in light-emitting diodes (LEDs). However, near-infrared (NIR) LEDs employing PQDs exhibit inferior external quantum efficiency related to the PQD emitting in the visible range. One fundamental issue arises from the PQDs dynamic surface: the ligand loss and ions migration to the interfacial sites serve as quenching centers, resulting in trap-assisted recombination and carrier loss. In this work, we developed a chemical treatment strategy to eliminate the interface quenching sites and achieve high carrier utilization. We employ a bidentate and liquid agent (Formamidine thiocyanate, FASCN) with tight binding to suppress the ligand loss and the formation of interfacial quenching sites: the FASCN-treated films exhibit fourfold higher binding energy than the original oleate ligands. Furthermore, the short ligands (carbon chain <3) enable the treated films to show eightfold higher conductivity; and the liquid characteristics of FASCN avoid the use of high polar solvents and guarantee better passivation. The high conductivity ensures efficient charge transportation, enabling PQD-based NIR-LEDs to have a record-low voltage of 1.6 V at 776 nm. Furthermore, the champion EQE of the treated LEDs is ~23%: this is twofold higher than the control, and represents the highest among reported PQD-based NIR-LEDs.

## Abstract (web summary)

This work employs a bidentate liquid agent to improve ligand coverage on QD surface, suppress ligand loss, and achieve a record ~23% EQE in target NIR-LEDs.

## Graphical abstract

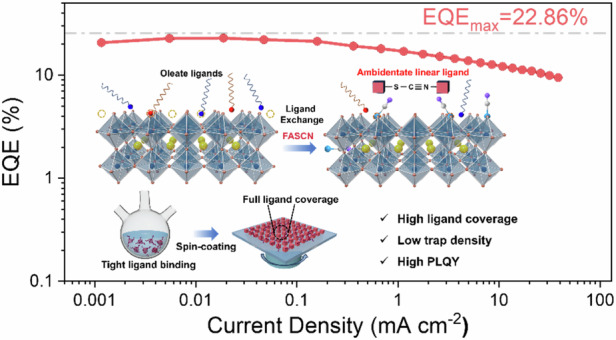

## Introduction

Quantum dots (QDs) are an ideal class of emitters for light-emitting diodes (LEDs)^1–3^. Metal-halide perovskite quantum dots (PQDs) unite structural diversity, bandgap tunability, color purity, and low-cost solution processing^4–7^. LEDs employing PQDs have shown external quantum efficiency (EQE) of over 20% in the visible range from 400 to 700 nm^8–10^. However, in the near-infrared I region (NIR-I, >750 nm), until now, the efficiency is still relatively low^11^.

**Fig. 1:**
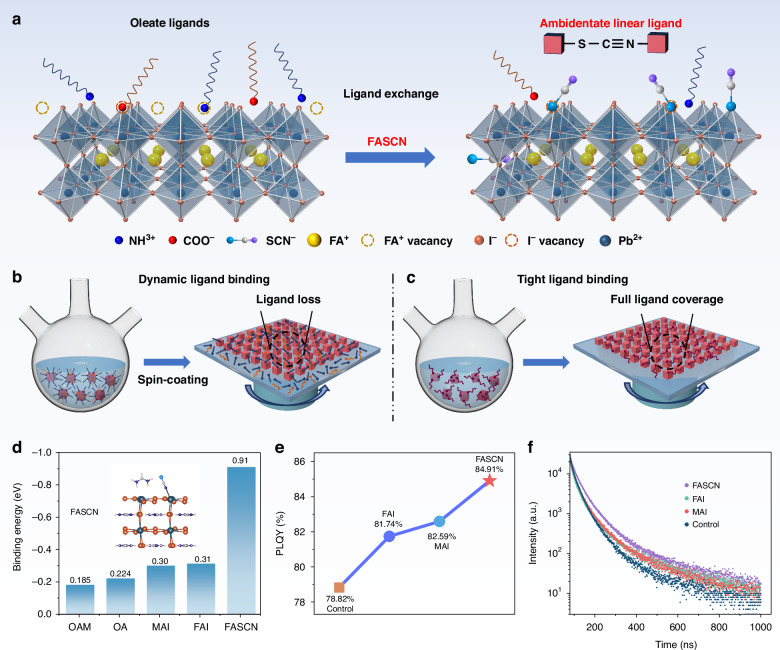
Mechanism and performance of ligand exchange. **a** Schematic illustrating oleate ligands and common halide ligands with incompact binding, and this work employed the bidentate and liquid reactive agent to enable full coverage and well-passivated QDs. **b** Schematic illustrating the dynamic ligand binding and **c** tight ligand binding on QDs surface during the film-preparation process. **d** Histogram of the binding energy to FAPbI_3_ QDs of various ligands. **e** PLQY and **f** TRPL spectra of FAPbI_3_ QDs after ligands exchange

The presence of surface and interfacial trap sites is the primary issue accounting for the inferior performance of PQD-based NIR-LEDs. Surface traps are formed by uncoordinated lead (Pb^2+^) during the synthesis. This is caused by the dynamic ligand binding of the oleate ligand and the surface^12–14^. The oleate ligands have long organic chains and form steric-repulsing interactions when capped on the QD surface, preventing full surface coverage. Ligand exchange employing short organic ligands without steric hindrance is an ideal way to improve the surface coverage and passivate the surface traps (Fig. 1a)^15–17^. However, the introduction of some short ligands would cause the occurrence of interfacial trap sites due to the incompact binding to the QD surface. Since most ligands provide high surface coverage only when present in excess in solution and have liable features, the ligands tend to desorbed from the surface and to the interfacial sites during the spin-coating process (Fig. 1b); these desorbed ions at interface the act as interfacial quenching centers, which deteriorate the device performance^18–20^.

We reasoned that this issue could be resolved only when the ligand has a short carbon chain to ensure high surface coverage, and at the same time, provide tight ligand-surface binding. We here employ a bidentate and liquid agent (Formamidine thiocyanate, FASCN) that satisfies these two requirements at the same time (Fig. 1c)^21–24^. The FASCN could form coordinate bonds simultaneously by using soft sulfur and nitrogen atoms and exhibits fourfold higher binding energy than the oleate ligands and threefold higher than FAI and MAI, preventing ligand desorption during the film-preparation process. The tight binding and short chain of FASCN ensures full coverage to the QD surface and improved conductivity of 3.95 × 10^−7^ S m^−1^, which is eightfold higher than the control, as evidenced by the two-terminal device. The FASCN-treated QD films exhibited an exciton binding energy of 76.3 meV, which is nearly twofold higher than the control films (39.1 meV). As a result, we fabricated PQD-based NIR-LEDs without carrier loss and achieved a recorded EQE of 23%, which is twofold higher than the control LEDs and represents the highest among reported PQD-based NIR-LEDs.

## Results

The inferior efficiency of PQD-based NIR-LEDs can be ascribed to the high trap density of QDs and dynamic surface ligands, which results in carrier loss and low mobility. Surface ligand exchange is widely recognized as an effective way to replenish the trap sites and replace the long oleate ligands, but the incompact ligand-QD binding usually leads to undesired ligand desorption and new trap sites during the film-preparation process, so the appropriate ligand is a prerequisite for high-performance QDs. We chose organic-inorganic hybrid FAPbI_3_ QDs to verify the advantage of the FASCN treatment and synthesized the FAPbI_3_ QDs with oleate acid (OA^−^) and oleylammonium (OAm^+^) capping ligands (Figs. S1, and S2). We treated the QDs with various ligands during the posttreatment. Initially, we calculated the binding energy (*E*_b_) of FASCN, oleate ligands (OA, OAm) and common halide ligands (FAI, MAI) on the host lattice FAPbI_3_ QDs through density-functional theory (DFT) (Figs. 1d, and S3). The *E*_b_ of FASCN (−0.91 eV) is fourfold larger than that of OAm (−0.18 eV) and OA (−0.22 eV), and is also visible higher than FAI (−0.31 eV) and MAI (−0.30 eV), indicating that the FASCN is thermodynamically more favorable for binding to the QDs surface. As shown in Fig. 1e, the photoluminescence quantum yield (PLQY) of QD got the most notable improvement after FASCN treatment, suggesting the effective passivation to surface trap sites, which was also demonstrated by the prolonged lifetime observed from time-resolved photoluminescence (TRPL) (Fig. 1f)^25,26^.

**Fig. 2:**
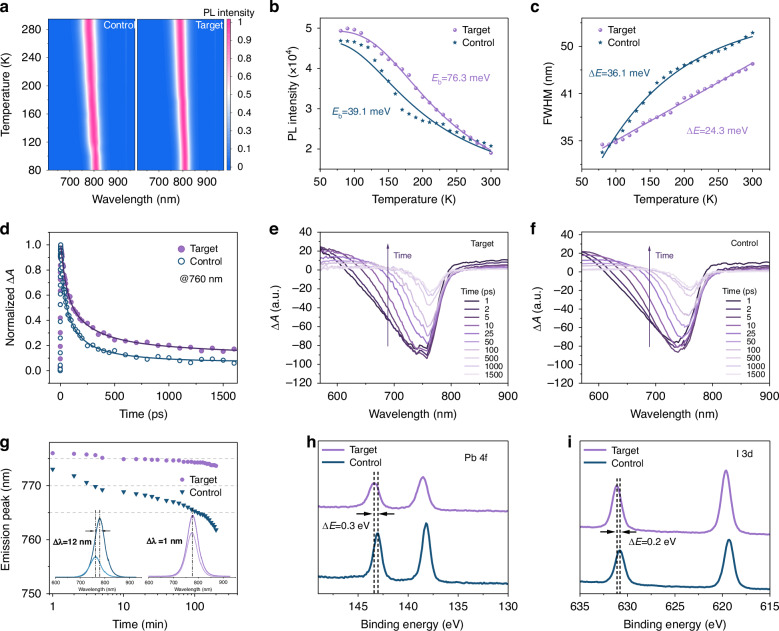
Spectroscopy characteristics of QDs. **a** Temperature-dependent PL spectra from 80 to 300 K for control and FASCN-treated FAPbI_3_ QD films. **b**, **c** Integrated PL emission intensity and the FWHM as a function of temperature for control and FASCN-treated FAPbI_3_ QD films. **d** Normalized decay kinetics of the photoinduced bleaching and **e**, **f** TA spectra at selected pump-probe delays for control and treated FAPbI_3_ QDs. **g** The emission peak dependence on aging time (Inset: initial PL spectra of the control and FASCN-treated QD films, and after 200 min). **h** Pb 4*f* and **i** I 3*d* core-level spectra of control and FASCN-treated QD films

We then performed temperature-dependent photoluminescence (PL) spectra to investigate the intrinsic characteristics of photogenerated excitons (Figs. 2a, and S4). We observed a notable increase in integrated PL intensity as the temperature dropped from 300 to 80 K attributed to decreased exciton dissociation and nonradiative trapping processes at lower temperatures (Fig. 2b). We calculated the exciton binding energy for each film and applied the Arrhenius equation to establish the functional relationship between the integrated PL emission intensity and inverse temperature^27^.

1 $$I\left(T\right)=\frac{{I}_{0}}{1+A{{\rm{e}}}^{-\frac{{E}_{b}}{{k}_{B}T}}}$$

Where *I*_*0*_ is the integrated PL intensity at 0 K, *E*_*b*_ is the exciton binding energy, *k*_*B*_ is the Boltzmann constant, and *A* is the coefficient. The *E*_*b*_ of FASCN-treated QD film is 76.3 meV, nearly twofold higher than control film (39.1 meV). The higher binding energy of the FASCN-treated film indicates a reduced chance of exciton dissociation into free carriers in the absence of radiation. The possibility of exciton nonradiative complexation decreases as the exciton binding energy increases.

The full width at half maximum (FWHM) of the PL spectra for the FASCN-treated film increased from 26.85 to 35.40 meV as the temperature rose from 80 to 300 K (Fig. 2c). Similarly, the FWHM of the control film increased from 23.56 to 36.23 meV. The reduction of structural vibration and deformation at lower temperatures helps to reduce electron-phonon coupling. To assess the strength of the electron-phonon coupling, we employed a fitting procedure to evaluate the temperature dependence of FWHM using the following equations:

2 $${\rm{FWHM}}\left({\rm{T}}\right)=\frac{{\Gamma }_{{LO}}}{\exp \left(\frac{{E}_{{LO}}}{{K}_{B}T}\right)-1}+C$$

Where E_*LO*_ represents the longitudinal optical (LO) phonon energy, and Г_*LO*_ signifies the coupling strength. The parameter *C* encompasses both the inhomogeneous broadening coefficient (Γinh) and the exciton-acoustic phonon coupling coefficient (σ). At low temperatures (<100 K), exciton-phonon interactions are dominated by acoustic phonons, and thermal diffusion has little effect on the system. Therefore, only factor C is considered in this regard. The fitting results demonstrate that the observed narrowing of the full width at half maximum (FWHM) can be explained by the decrease in LO phonon coupling^28^.

To further study the charge transfer and recombination dynamics of photons after generation, we employed femtosecond transient absorption (TA) spectroscopy (190 fs pulse width, 450 nm excited, 10 mW). The TA spectra of QDs are illustrated in Fig. S5, with probes ranging from 550 to 900 nm and scan delay times of 0.2 ps to 1 ns. We observed a strong ground bleaching signal centered at 750 nm, and the bleaching agent recovered within about 1 ps, indicating a rapid transfer of charge from the excited to the emissive state (Fig. 2d–f)^29^. The absorption feature decreased more rapidly after FASCN treatment, suggesting the introduction of additional energy carrier transfer and recombination pathways during the treatment^30^. In addition, we observed a consistent bleaching peak with an unsharp shift of FASCN-treated QDs.

We also assessed the thermal stability of the QD films under continuous heating at 100 °C by recording the PL intensity and the emission wavelength as a function of time, and found that the control films exhibited severe thermal quenching (Fig. 2g). We observed the FASCN-treated films exhibited good thermal stability with no observable emission shift with increasing heating time (Δ*λ* = 1 nm), while the control films suffered from a significant shift with a Δ*λ* of 12 nm, and the improved thermal stability was also clearly revealed by the PL intensity map (Fig. S6). In addition to thermal stability, we also investigated the humidity stability by exposing the QD films to humidity >99% (Fig. S7). After 30 min, the control film had corroded heavily while the target film was still intact, indicating the full ligand coverage and more compact QD structure ensure better water-oxygen stability. Then, we performed X-ray diffraction (XRD) of QD films after annealing over time to investigate the phase stability (Fig. S8), we observed a more obvious peak intensity attenuation of the control film than that of the target film during 2 h of annealing.

We also performed X-ray photoelectron spectroscopy (XPS) to further investigate the mechanism of FASCN treatment on QDs surface. In Fig. 2h, the Pb 4*f* peak position of the FASCN-treated sample is shifted to higher binding energy, suggesting the environmental composition changes and the electron density around Pb^2+^ increases with the introduction of FASCN. In addition, the I 3*d* peak position shifts 0.2 eV to the higher binding energy of the treated films, indicating the passivation of I vacancies (Fig. 2i)^31^. We also monitored the presence of the S 2*p* peak on QDs before and after ligand exchange (Fig. S9). The distinct S 2*p* peak was observed on FASCN-treated QDs, indicating that the FASCN ligand was successfully induced into the QDs surface^23^. The existence of the SCN^−^ around QDs can also be verified by Fourier-transform infrared spectroscopy (FTIR), where the characteristic peak at 2248 cm^−1^ can be assigned to the stretching vibration of triple bonds from SCN^−^ (Fig. S10)^32^. In addition, we observed the decreased intensity of FTIR peaks for FASCN-treated QD films, indicating the oleate ligands were further replaced. In addition, the ligand density decreases when increasing the FASCN concentration, the result can also be observed in ^1^H nuclear magnetic resonance spectra (Fig. S11). The atomic force microscopy (AFM) height images (Fig. S12) suggest that the FASCN-treated QDs films produce more homogeneous surface morphology. The root-mean-square roughness value of the FASCN-treated film is 3.77 nm, which is 1.9-fold smaller than that of the control one (7.36 nm).

**Fig. 3:**
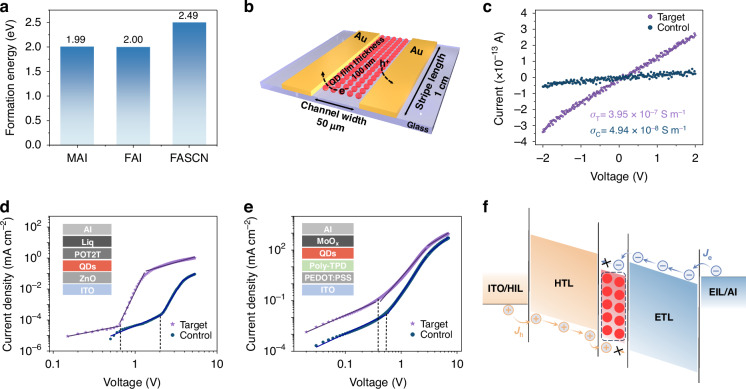
Trap and mobility analysis of QD films. **a** Histogram of the defect formation energy of the iodide vacancy. **b** Schematic diagram of the two-terminal device. **c** The I–V curve of control and target two-terminal devices. **d**, **e** Space charge-limited current for electron-only and hole-only devices. **f** Schematic illustration of the carrier dynamic in NIR-LEDs

In addition to the strong binding of the ligand to the QD surface, we also calculated the formation energy of the iodide vacancy (*I*_v_). The formation energies for QDs treated with MAI, FAI, and FASCN are 1.99 eV, 2.00 eV, and 2.49 eV, respectively. These results indicate that FASCN exhibits superior defect passivation ability, which effectively reduces the density of traps and, consequently, enhances the conductivity of the QD films treated by FASCN (Fig. 3a). We then examine the conductivity of QD films through a two-terminal device (Fig. 3b). The resulting current-voltage (I-V) curve of the FASCN-treated films exhibited a notable increase in slope compared to the control films. Calculating from the I-V slope, with fixed channel length and width, the FASCN-treated films showed a conductivity (*σ*) of 3.95 × 10^−7^ S m^−1^, which is nearly eightfold higher than the control (4.94 × 10^−8^ S m^−1^), indicating the removal of surface insulting ligands (Fig. 3c)^33^. For the out-of-plane mobility, we built electron-only and hole-only devices to study the electrical transport mobility of QD films using the space charge limited current (SCLC) method. The current density-voltage (J-V) curves of electron-only and hole-only devices exhibit three modes: Ohmic contact, trap-filling limit, and child region (Fig. 3d, e). By fitting the child region and integrating the fit using the Mott–Gurney equation, the charge mobility (*μ*) of QD films can be obtained using the following equation (Tables S2, and 3)^34,35^:

3 $$\mu =\frac{8J{d}^{3}}{9{\varepsilon }_{r}{\varepsilon }_{0}{V}^{2}}$$

Where *J*, *d*, and *V* are the dark current density measured in the SCLC region, the thickness of QDs films, and applied voltage, respectively. *ε*_0_ is the vacuum permittivity (*ε*_0_ = 8.854 × 10^−14 ^F cm^−1^), *ε*_*r*_ is the relative permittivity of perovskite (*ε*_*r*_ = 46.9). We obtained electron mobility of 4.2 × 10^−4 ^cm^2 ^V^−1^ s^−1^ and hole mobility of 1.01 × 10^−3 ^cm^2 ^V^−1^ s^−1^ for the target device, which is 4.8-fold/2.3-fold higher than control device (8.67 × 10^−5 ^cm^2 ^V^−1^ s^−1^ and 4.29 × 10^−4 ^cm^2 ^V^−1^ s^−1^). Moreover, the smaller difference for FASCN-treated films between electron and hole mobility also suggests a more balanced carrier recombination, especially when these films were utilized in LEDs (Fig. 3f).

We also investigated the trap density of the QD films after FASCN treatment. The electron-only devices present trap-filling behavior at a lower voltage, a metric we refer to as trap-filled limit voltage (*V*_*TFL*_). We compared the trap density (*n*_*trap*_) of the QD films using the following equation:

4 $${n}_{{trap}}=\frac{2{\varepsilon }_{r}{\varepsilon }_{0}{V}_{{TFL}}}{e{d}^{2}}$$

Where *V*_*TFL*_ is the trap-filled limit voltage, *e* is the elemental charge, and *d* is film thickness^36^. The estimated trap density of FASCN-treated QD films is 2.95 × 10^−17 ^cm^−3^, which is threefold lower than that of control films, indicating the significant passivation effect of FASCN treatment.

**Fig. 4:**
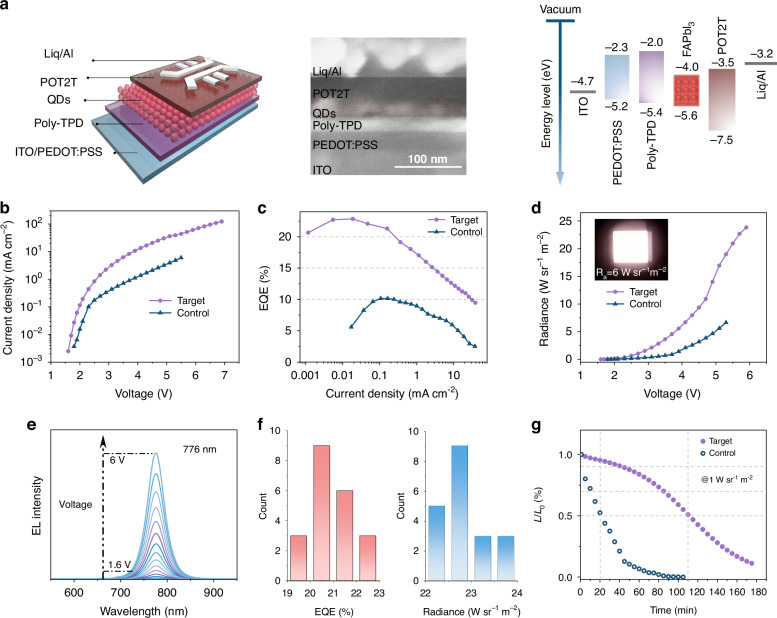
Device performance of FASCN-treated NIR-LEDs. **a** Energy level diagram of each layer and cross-sectional SEM image of FASCN-treated NIR-LEDs. The energy levels of the FAPbI_3_ QDs were calculated from ultraviolet photoelectron spectroscopy (**UPS** in Fig. S13). **b** Current density-voltage curves, **c** EQE-current density curves and **d** radiance-voltage curves of control and FASCN-treated NIR-LEDs. **e** EL spectra from 1.6 V to 6 V and **f** peak EQE and radiance histogram of FASCN-treated NIR-LEDs. **g** Operational stability measurement of control and FASCN-treated NIR-LEDs at an initial radiance of 1 W sr^−1^ m^−2^ at ambient environment

Encouraged by the combined high PLQY and high carrier mobility, we employed FASCN-treated QD films as emitters to fabricate the NIR-LEDs. We built a device configuration of indium tin oxide (ITO)/(PEDOT:PSS)/poly[N,N’bis(4-butylphenyl)-N,N’-bis(phenyl)-benzidine](poly-TPD)/QDs/(1,3,5-triazine-2,4,6-triyl)tris(benzene-3,1-diyl) tris (diphenylp-hosphine oxide)(PO-T2T)/lithium 8-quinolate (Liq)/Al, and the cross-sectional scanning electron microscopy (SEM) image shows each corresponding layer (Fig. 4a).

The LED-based on FASCN-treated FAPbI_3_ QDs displayed a low turn-on voltage of 1.6 V. The NIR-LEDs showed a peak radiance of 24 W sr^−1^ m^−2^, representing a fourfold improvement compared to the control LEDs (Fig. 4d). The larger current density observed, compared to the control device, is attributed to higher conductivity, stemming from the decrease of trap density in the target LEDs. The electroluminescence (EL) spectra of these NIR-LEDs reveal a symmetrical EL peak at 776 nm with a full width at half maximum (FWHM) of 45 nm, importantly, the spectra remain constant within the voltage range of 1.6 to 6 V (Fig. 4e). The FASCN-treated LEDs achieved a champion EQE of 22.86%, which is nearly twofold higher than the control, and represents the highest among reported PQD-based NIR-LEDs (Fig. 4c). Furthermore, FASCN-treated LEDs exhibited consistent reproducibility, we fabricated twenty LEDs using identical procedures, and their peak EQE and peak radiance histograms are presented in Fig. 4f, revealing a mean EQE of 20.7% with a minimal standard deviation of 0.15% and a mean radiance of 22.75 W sr^−1^ m^−2^. We recorded the stability of LEDs at an initial radiance of 1 W sr^−1^ m^−2^ in the ambient environment (Fig. 4g), the FASCN-treated LEDs exhibited a half-life (*T*_50_) of 115 min, which is nearly fivefold higher than the control.

## Discussion

In summary, we employed a bidentate and liquid agent (FASCN) that ensures high surface coverage and provides tight ligand-surface binding on the QDs surface, and the liquid characteristics of FASCN avoid the use of high polar solvents and guarantee better passivation. This strategy enabled us to fabricate trap-free perovskite QD films with higher conductivity, stability, and exciton binding energy. Implementing this strategy, we reported the PQD-based NIR-LEDs at 776 nm with an EQE of ≈23%, which is twofold higher than the control, and represents the highest among reported PQD-based NIR-LEDs. This chemical treatment strategy will contribute to much-needed further progress in resurfacing PQD films for NIR LEDs.

## Materials and methods

### Materials

Lead (II) iodide (PbI_2_, 99%, Sigma-Aldrich), Formamidine acetate (HN=CHNH_2_·CH_3_COOH, 99%, Alfa Aesar), Oleylamine (OAm, technical grade 70%, Sigma-Aldrich), Oleic acid (OA, technical grade 90%, Sigma-Aldrich), 1-Octadecene (ODE, technical grade 90%, Adamas), Methyl acetate (Reagent Plus, 99%, Sigma-Aldrich), Octane (anhydrous, ≥99%, Sigma-Aldrich). PEDOT: PSS solution (Clevios PVP AI 4083), poly-TPD, and PO-T2T were purchased from Xi’an Polymer Light Technology Corp. All the chemicals were used directly as received.

### Synthesis of FAPbI_3_ QDs

In a typical synthesis, 650 mg FA(AC), and 6.0 ml OA were stirred in a 3-necked round bottom flask at room temperature for 5 min. The reaction mixture was degassed for 5 min at room temperature rapidly heated to 140 °C and continued to be degassed for 120 min. After the reaction form a clear FA-oleate precursor solution. Nitrogen (N_2_) was introduced, and the temperature was kept at 120 °C. Before hot injection, the system temperature of the FA-precursor was decreased to 100 °C.

In another flask, 350 mg PbI_2_ was mixed with 2 mL of OAm/OA mixture and 20 mL of ODE followed by vacuum drying at 100 °C for 100 min. Under the N_2_ atmosphere, the lead halide precursors were kept at a reaction temperature of 80 °C until all solids were dissolved. Then, 4.0 mL FA-precursor solution was rapidly injected into the flask containing the lead halide precursor. The solution was then swiftly cooled using an ice water bath. The solution was centrifuged at 7800 rpm to remove unreacted precursors. The supernatant was discarded, and the precipitation was collected. Then dissolve the precipitate with octane. During the two-step purification process, methyl acetate was added to the QDs solution. The mixture was then centrifuged to collect the precipitant and dissolve in the octane. The crude solution was centrifuged at 7800 rpm to remove unreacted precursors. The supernatant was discarded, and the precipitation was collected. Then dissolve the precipitate with octane to obtain FAPbI_3_ crude QDs solution.

### Two-step purification strategy

Methyl acetate was added to the crude QDs solution. The mixture was then centrifuged to collect the precipitant and dissolve in the octane. Then add methyl acetate into the solution and centrifuge again. Collect the precipitant and dissolve it in the octane to obtain a purified FAPbI_3_ solution. The QDs solution was stored at 4 °C until further use.

### FASCN ligand exchange strategy

During the second purification process, replace the methyl acetate with the 0.1 mg mL^−1^ FASCN solution (dissolved in methyl acetate) and centrifuge. Collect the precipitant and dissolve it in the octane to obtain FASCN-treated FAPbI_3_ QDs solution. The QDs solution was stored at 4 °C until further use.

### FAPbI_3_-based NIR-LEDs fabrication

For fabricating FAPbI_3_ - based NIR-LEDs, ITO/glass substrates were cleaned using deionized water, acetone, and 2-propanol ultrasonically for 60, 60, and 60 min, respectively. After this, the substrate was dried with nitrogen flow and treated with UV-ozone for 15 min. Then, PEDOT: PSS was spin-coated at 4000 rpm for 30 s, followed by annealing on a hotplate at 150 °C for 15 min. After the substrate was cooled to room temperature, a solution of 8 mg mL^−1^ poly-TPD chlorobenzene was spin-coated onto the PEDOT: PSS layer at 4000 rpm for 40 s and bake for 15 min at 100 °C under a nitrogen atmosphere. FAPbI_3_ QDs were then spin-coated onto the Poly-TPD at 3000 rpm for 40 s and anneal for 60 to evaporate the solvent. Finally, ~60 nm of POT2T, ~2 nm of Liq and ~80 nm of Al were deposited using a thermal evaporation system (Suzhou Fang sheng FS380-S12) through shadow masks under a high vacuum (<10^−4^ Pa). Deposited using a thermal evaporation system (Suzhou Fang sheng FS380-S12) through shadow masks under a high vacuum (<10^−4^ Pa).

### Characterization of QDs

UV-vis absorption spectra were measured by a Shimadzu UV-3600PC scanning spectrophotometer. The photoluminescence (PL) spectra were collected by a fluorescence spectrometer (NIR-VIS, FL3). PL decay spectra were obtained by using the PL-TCSPC fluorescence lifetime measurement system (C12132-38, Hamamatsu Photonics Co.). The FTIR spectra were measured by VERTEX 70 FT-IR Spectrometer under transmission mode. The samples were prepared by dripping the solution into KBr powder and pressed in powder compaction presses. Transient PL decays were measured by HORIB-FM-2015. 1H NMR spectra were measured by utilizing the JNM-ECZ400S/L1 with a frequency of 400 MHz. XRD patterns were measured using a Bruker AXS D8 diffractometer with Cu Kα radiation (*λ* = 1.54178 Å). TEM overview images were taken through a Talos F200X electron microscope operated at 200 kV and analyzed through the Velox software. The UPS and XPS measurements were conducted using KRATOS AXIS Ultra DLD with a base pressure of ~10^−9^ torr. Atomic force microscopy (AFM) measurements were operated by using a Cypher-S atomic force microscope (Asylum Research, Oxford Instruments, UK).

### Characterization of PQD-based NIR-LEDs

Current density–voltage (J–V) characteristics were monitored by using a computer-controlled Keithley 2400 source meter. Electroluminescence (EL) spectra and external quantum efficiency (EQE) were tested by using a calibrated photonic multichannel PMA-12 analyzer system (Hamamatsu C10027-01 (360–950 nm) and C10028-01 (950–1600 nm)). The PMA-12 system was connected to an integrating sphere (3.3 in, collecting device forward light) and a power supply system (controlling current output) while a PR-745 instrument (Photo Research, 380–1060 nm) was used to calibrate the absolute radiance. All the measurements assumed Lambertian emission. All tests were conducted under ambient air conditions (*T* = 25–30 °C, *H* = 50–60%). During the lifetime test, the initial radiance was consistently set at 1 W sr^−1 ^m^−2^, with the radiance value recorded every 5 min to track the performance over time.

### Density functional theory calculations

Density functional theory (DFT) as implemented in the Vienna ab initio simulation package (VASP), was used to carry out the calculations presented here. The projector augmented wave (PAW) method was used to treat the effective interaction of the core electrons and nucleus with the valence electrons, while exchange and correlation were described using the Perdew–Burke–Ernzerhof (PBE) functional. The cut-off energy is set at 400 eV for the plane-wave basis restriction in all calculations. K-points are sampled under the Monkhorst–Pack scheme for the Brillouin-zone integration (K-points were sampled using the Gamma Point). In all calculations, the forces acting on all atoms are <0.02 eV Å^−1^ in fully relaxed structures, and self-consistency accuracy of 10^−5^ eV is reached for electronic loops. The binding energy was calculated as follows:

5 $${E}_{{binding}}=E-{E}_{a}-{E}_{b}$$

Where *E* is the total energy of the adsorbed system, *E*_a_ and *E*_b_ represent the total energy of tree species and bare surface, respectively.

### Two-terminal device calculations

The conductivity (*σ*) of the quantum dot films has been calculated using the parameters from the Two-Terminal Device. Specifically, it is derived from the length (*L*), width (*W*), and thickness (*T*) of the light-emitting layer, along with the slope (*S*) obtained from the I-V curve. The calculation formula is as follows:

6 $$\sigma =\frac{{SL}}{{WT}}$$

### Encapsulation technique and storage condition of LEDs

We encapsulated LEDs in a glovebox using a glass encapsulation cover and UV-curable adhesive to protect the device structure. The LED devices were stored in the glovebox under N_2_ (H_2_O and O_2_ < 0.1 ppm) at a temperature between 25 °C and 30 °C, with humidity levels maintained between 15 and 20%.

## Supplementary information

Supplementary Information

## Data Availability

The data that supports the plots within this paper and other findings of this study are available from the corresponding author upon reasonable request.
