# Supplementary material for: Liquid bidentate ligand for full ligand coverage towards efficient near-infrared perovskite quantum dot LEDs
Source: Light Sci Appl. 2025 Jan 7;14:35. doi: 10.1038/s41377-024-01704-x (PMC11704330; doi:10.1038/s41377-024-01704-x)
Supplement: Supplementary file 1 — Supplementary Information [file 41377_2024_1704_MOESM1_ESM.docx]

Supplementary Information for

**Liquid bidentate ligand for full ligand coverage towards efficient near-infrared perovskite quantum dot LEDs**

Zong-Shuo Liu^1,#^, Ye Wang^1,#^, Feng Zhao^1,#^, Hua-Hui Li^1^, Wei-Zhi Liu^1^, Wan-Shan Shen^1^, Hong-Wei Duan^1^, Ya-Kun Wang^1,*^, and Liang-Sheng Liao^1,2,*^

^1^ *Institute of Functional Nano & Soft Materials (FUNSOM), Jiangsu Key Laboratory for Carbon-Based Functional Materials & Devices, Soochow University, Suzhou 215123, China*

*^2^* *Macao Institute of Materials Science and Engineering, Macau University of Science and Technology, Macau SAR, Taipa 999078, China*

**Contents**

**1. Supporting Tables**

**2. Supporting Figures**

1. **Supplementary Tables**

| **Sample** | **τ_ave_ (ns)** | **τ_1_(ns)** | **τ_2_** | **τ_3_** |
| --- | --- | --- | --- | --- |
| **Target** | 63.08 | 15.63 | 52.80 | 246.79 |
| **MAI** | 52.26 | 10.78 | 42.14 | 155.09 |
| **FAI** | 50.49 | 10.65 | 40.02 | 154.11 |
| **Control** | 41.05 | 10.41 | 37.68 | 155.09 |

**Table. S1** The detailed parameters of TRPL spectra of FAPbI_3_ QD films.

**Table. S2** The thickness of each layer in the hole-only devices.

| **Hole-only** | **1** | **2** | **3** | **4** | **5** | **6** | **Average（nm）** |
| --- | --- | --- | --- | --- | --- | --- | --- |
| **HTL** | 30.37 | 31.54 | 33.01 | 33.11 | 33.77 | 33.93 | **32.62** |
| **QDs** | 107.50 | 107.75 | 107.84 | 105.87 | 108.12 | 108.43 | **107.59** |

| **Electron-only** | **1** | **2** | **3** | **4** | **5** | **6** | **Average（nm）** |
| --- | --- | --- | --- | --- | --- | --- | --- |
| **ETL** | 23.56 | 23.95 | 24.13 | 24.38 | 24.41 | 24.56 | **24.16** |
| **QDs** | 106.48 | 106.92 | 107.1 | 107.17 | 107.4 | 107.44 | **107.09** |

**Table. S3** The thickness of each layer in the electron-only devices.

**2. Supplementary Figures**


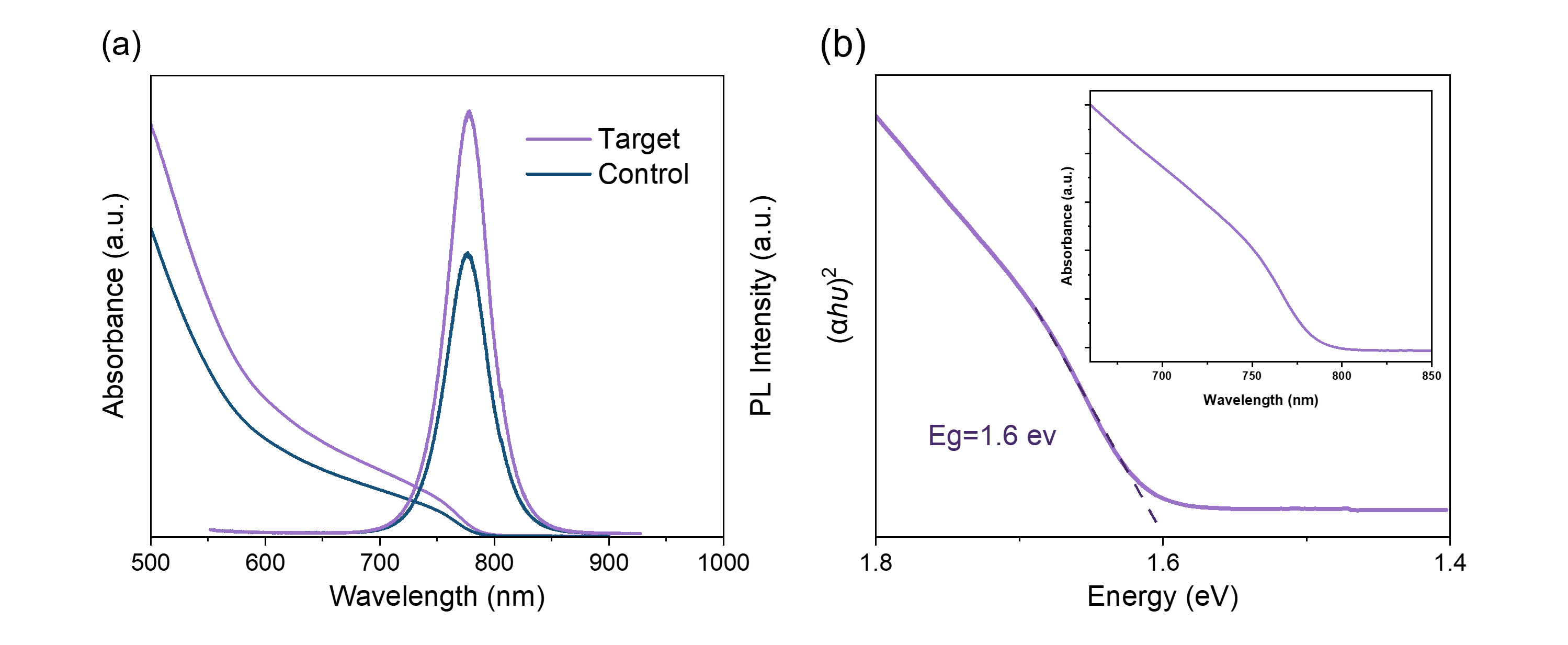


**Fig. S1.** (a) The UV-vis absorption and steady-state PL spectrum of control and target QDs. (b) The tauc plot of target QDs.


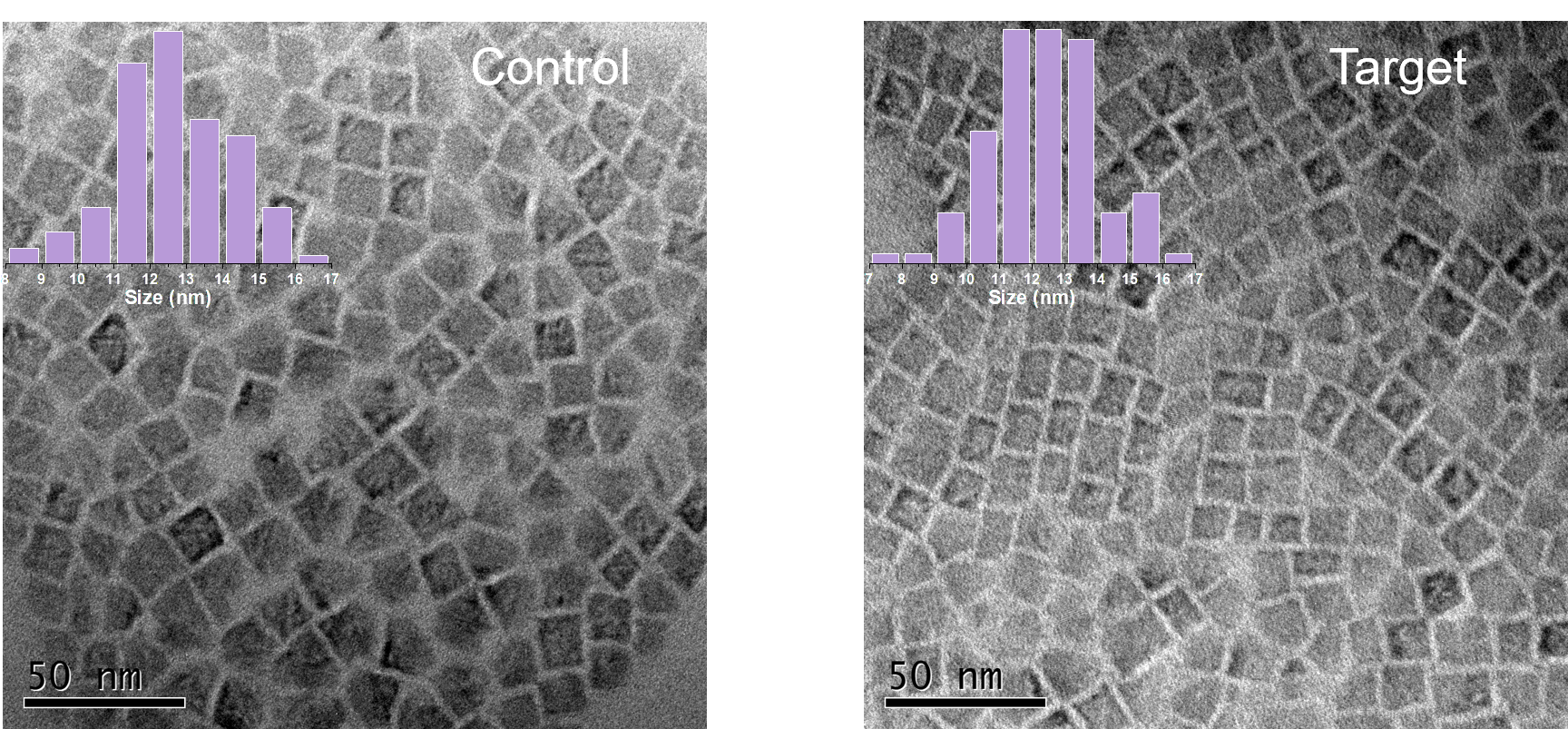


**Fig. S2.** TEM images of control and target QDs.


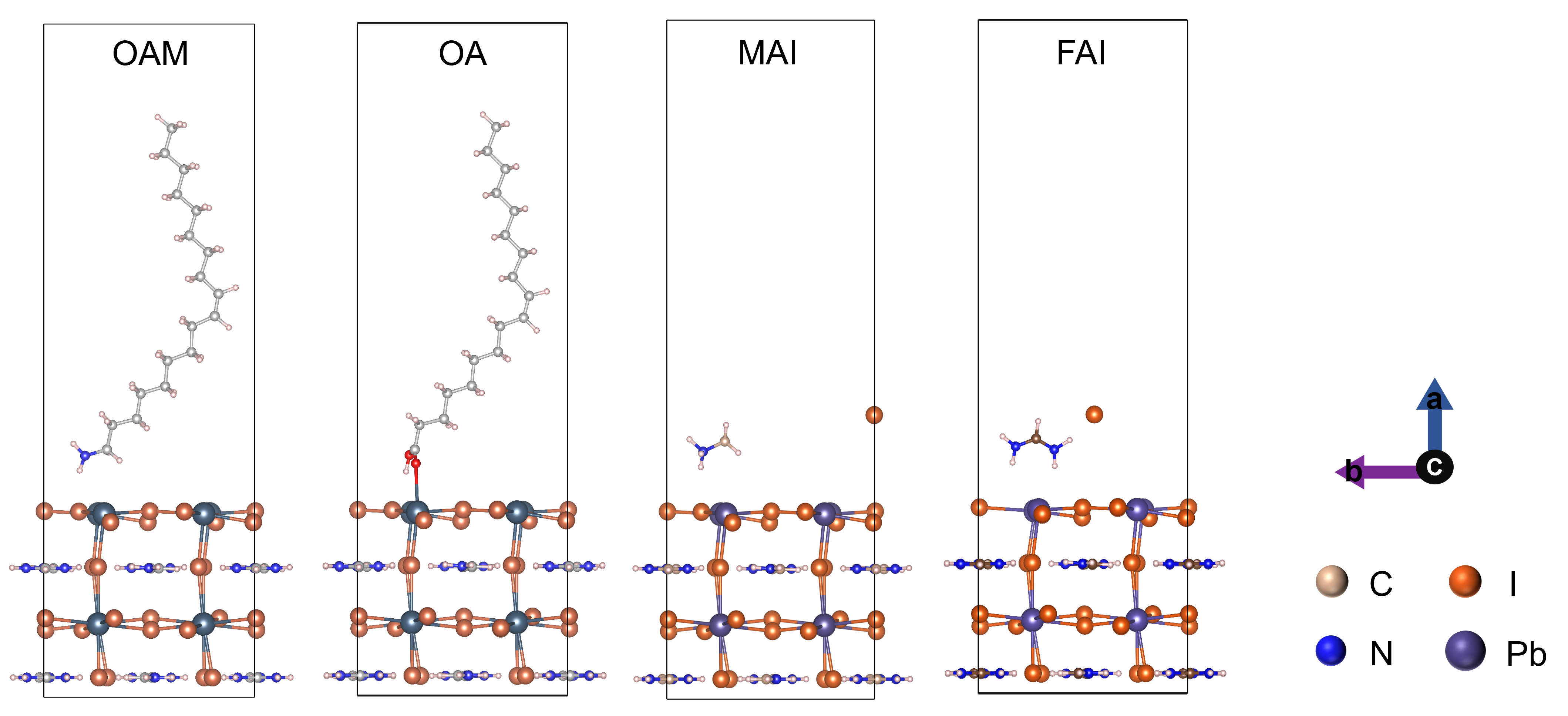


**Fig. S3.** Theoretical calculations of the binding energy of different ligands on the surface of the FAPbI_3_ QDs.


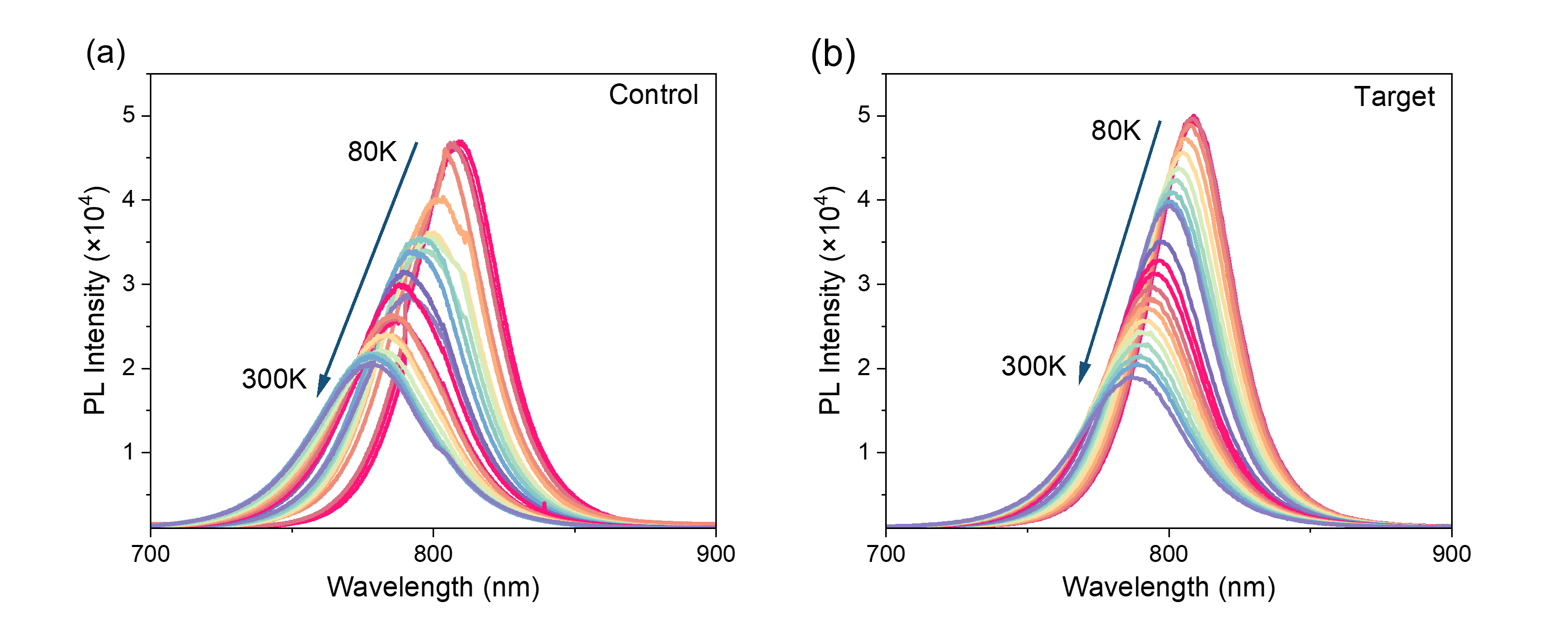


**Fig. S4.** Temperature-dependent PL characterization. PL spectra from 80 to 300 K for (a) control QD film and (b) target QD film.


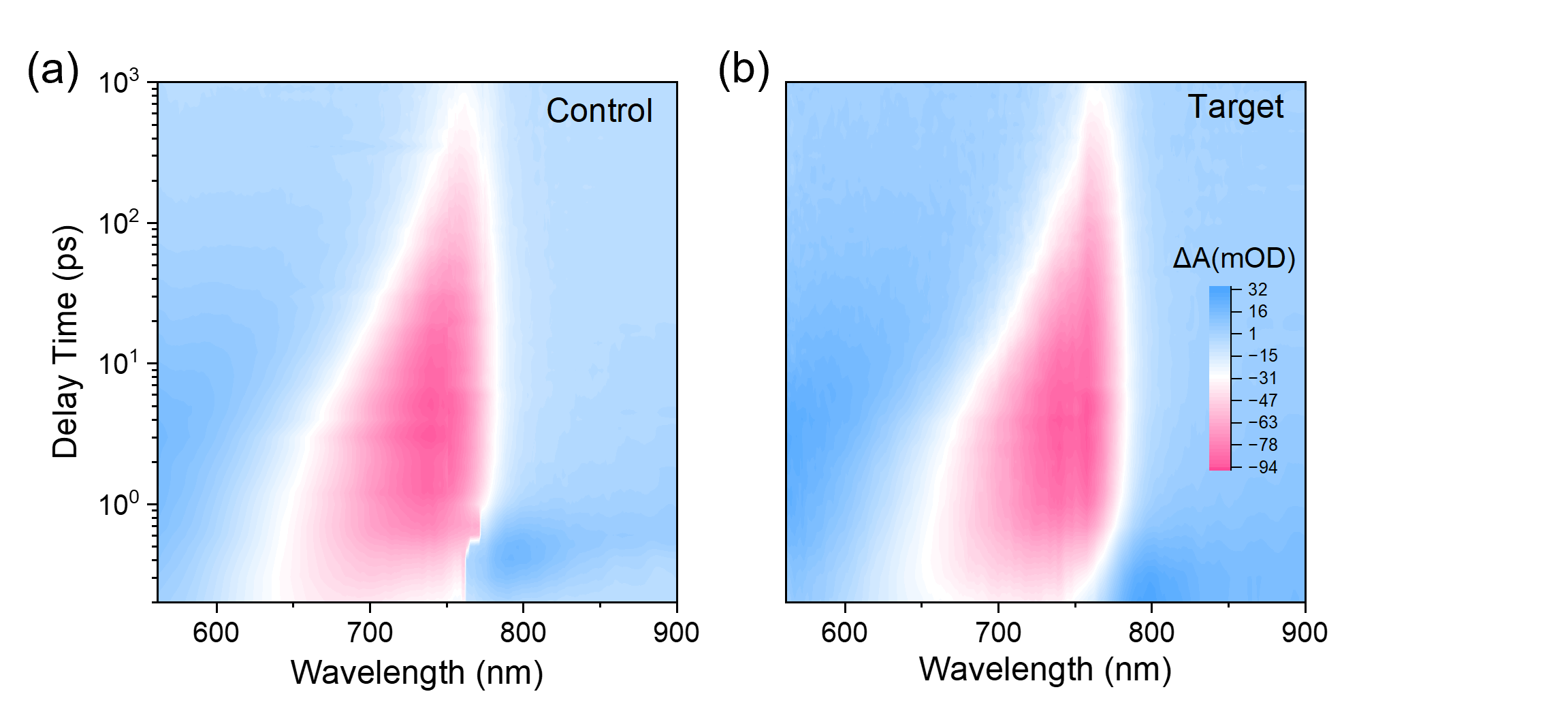


**Fig. S5.** Pseudo-colour TA maps of (a) control (b) target FAPbI_3_ QDs.


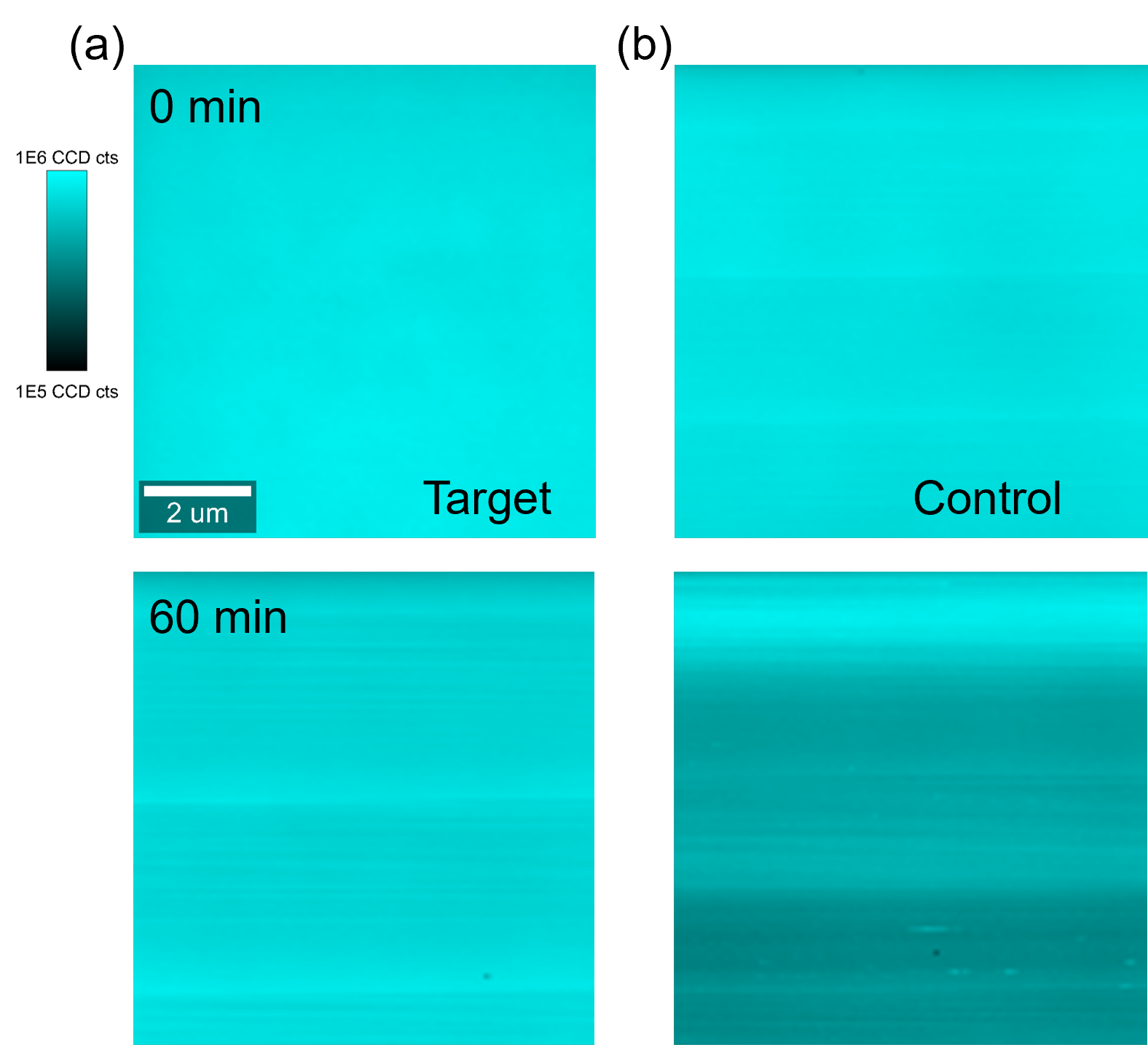


**Fig. S6** PL intensity map of (a) control and (b) target QD films heating at 60 ℃ for different times (0, 60 min).


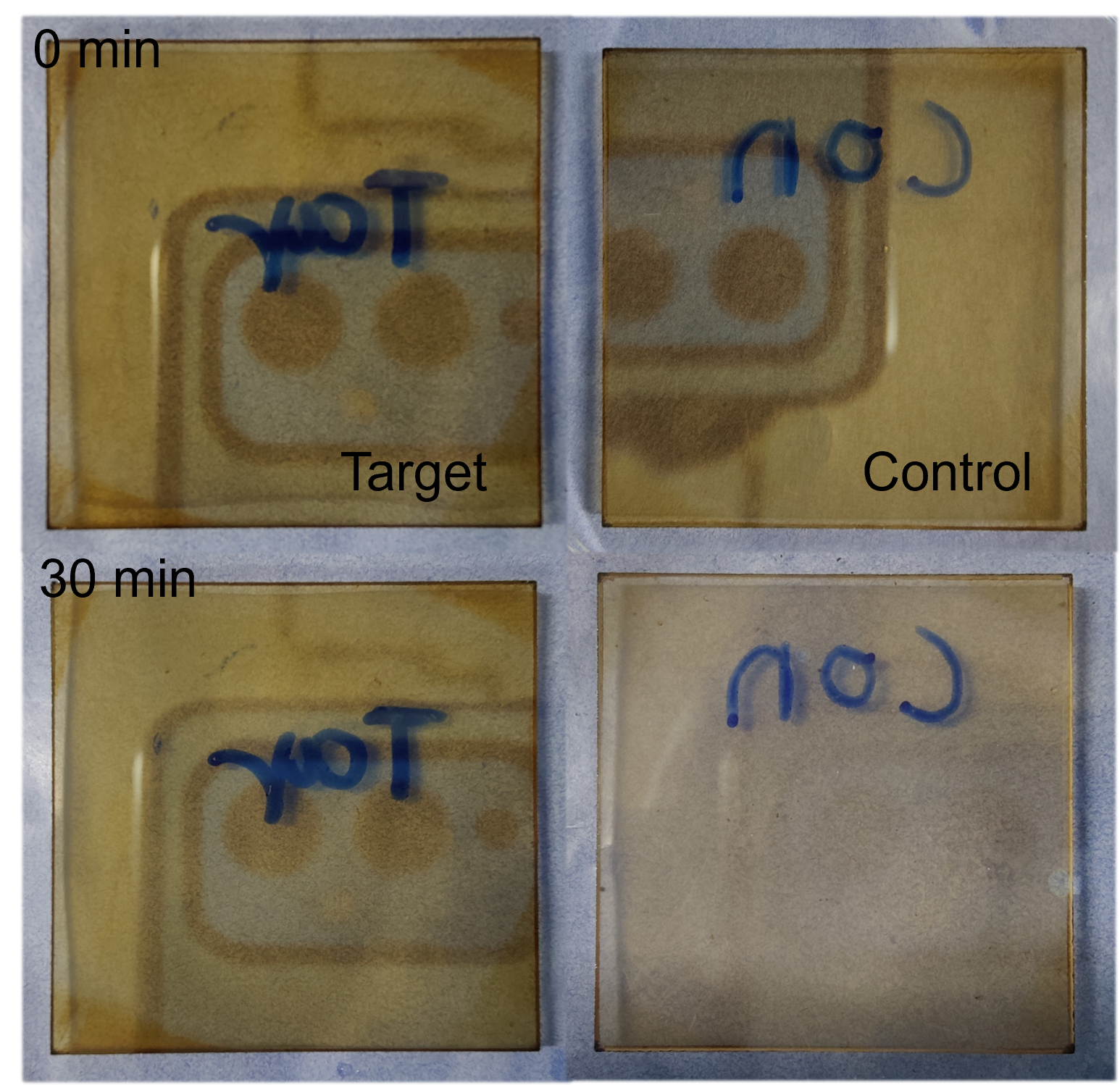


**Fig. S7.** Photos of control and target QD films exposed to humidity of > 99% over time.


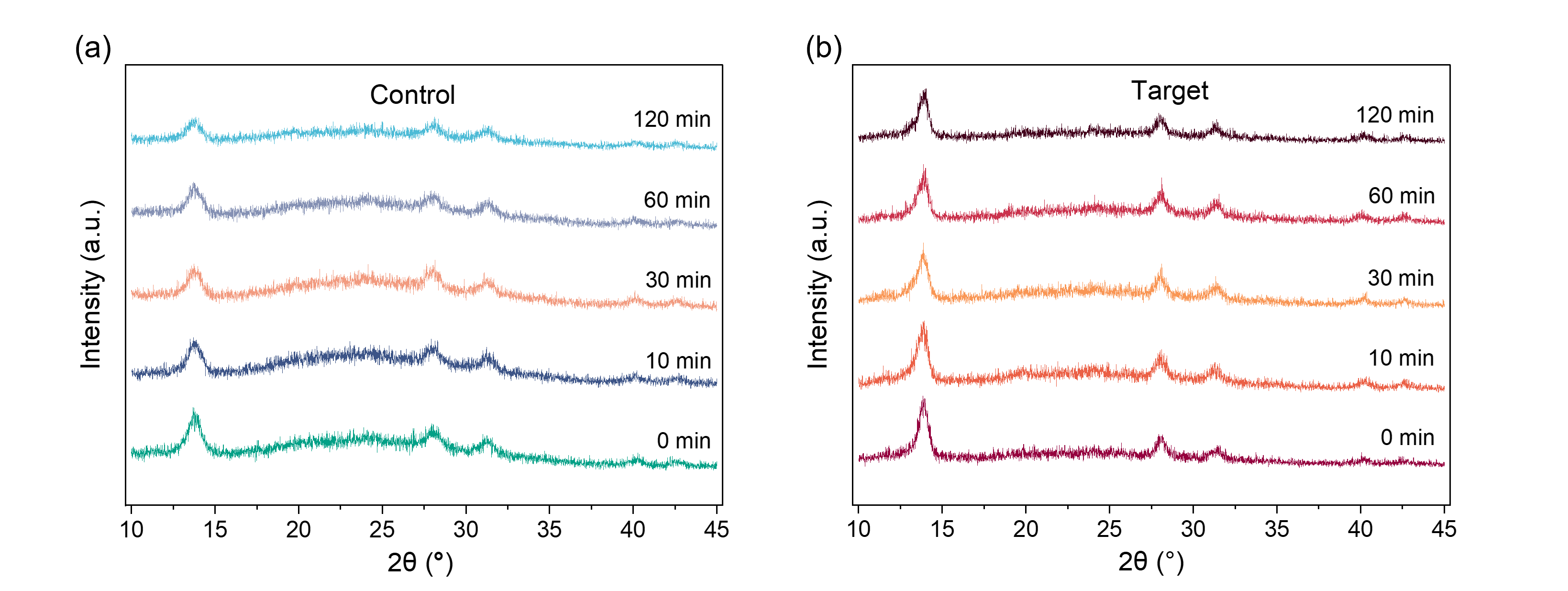


**Fig. S8.** XRD spectra of control and target FAPbI_3_ QDs after annealing at 80 ℃ for different times (0, 10, 30, 60, 120 min).


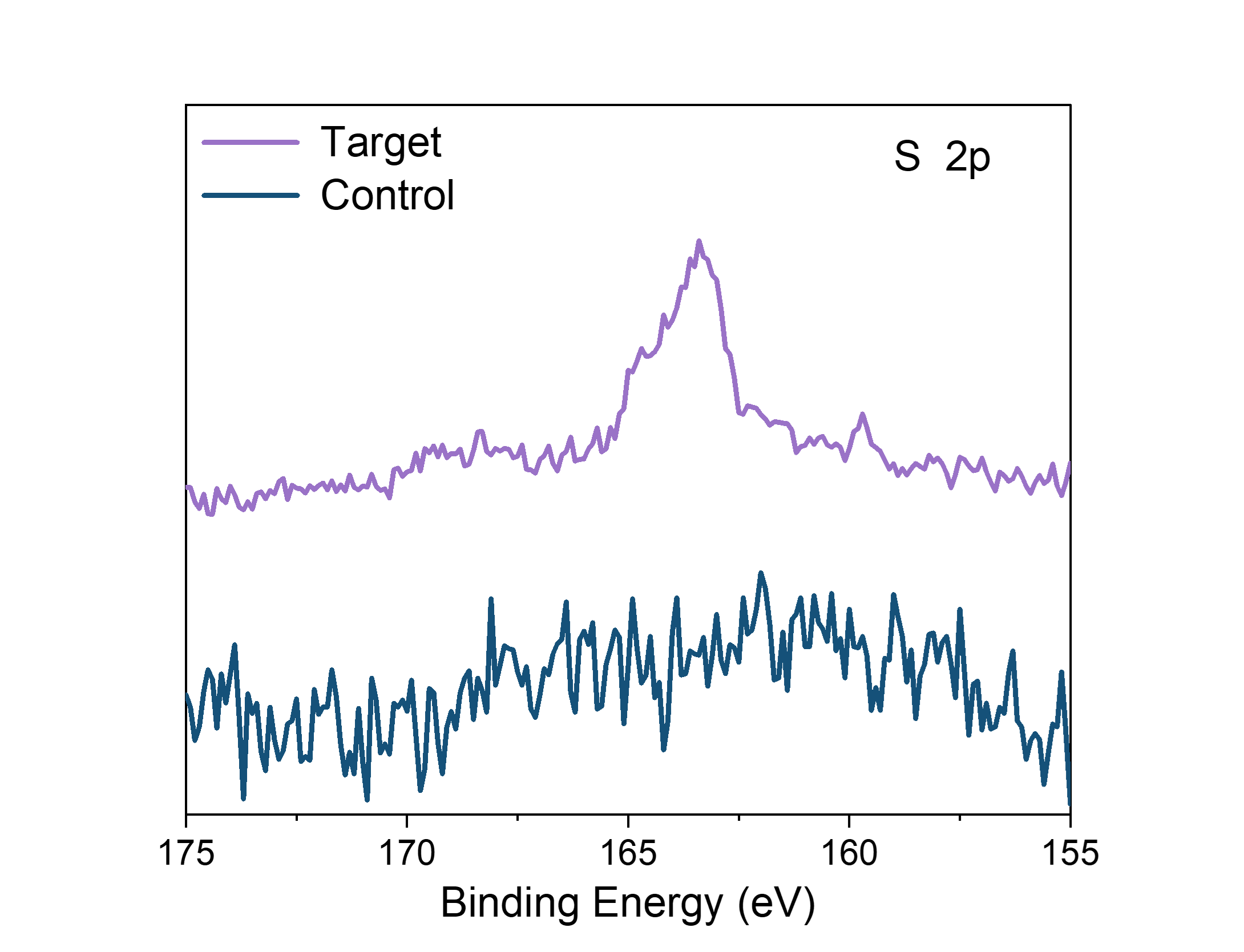


**Fig. S9.** S 2p core-level spectra of control and target QD films.


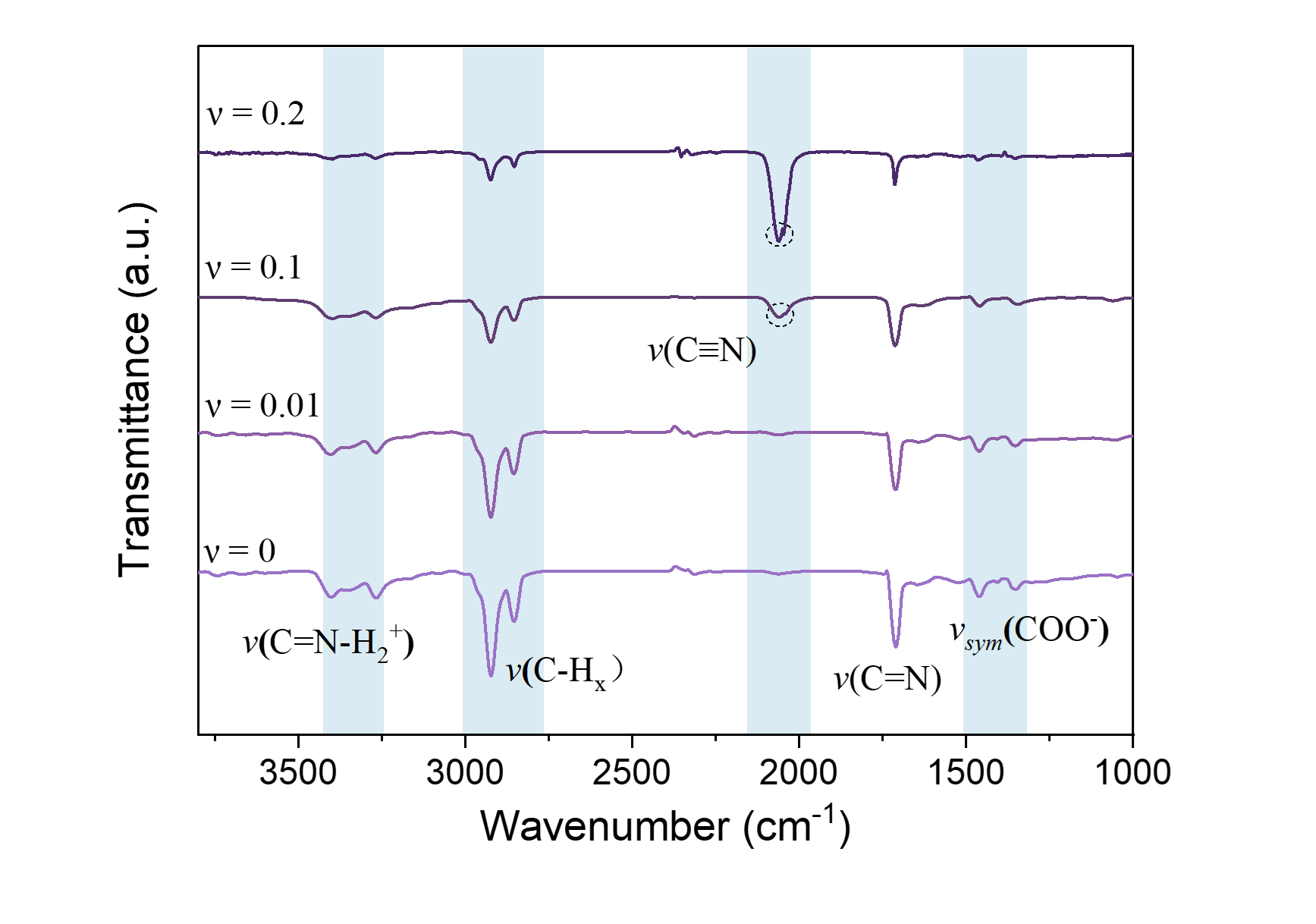


**Fig. S10.** Fourier-transform infrared spectroscopy of control and target QD films.


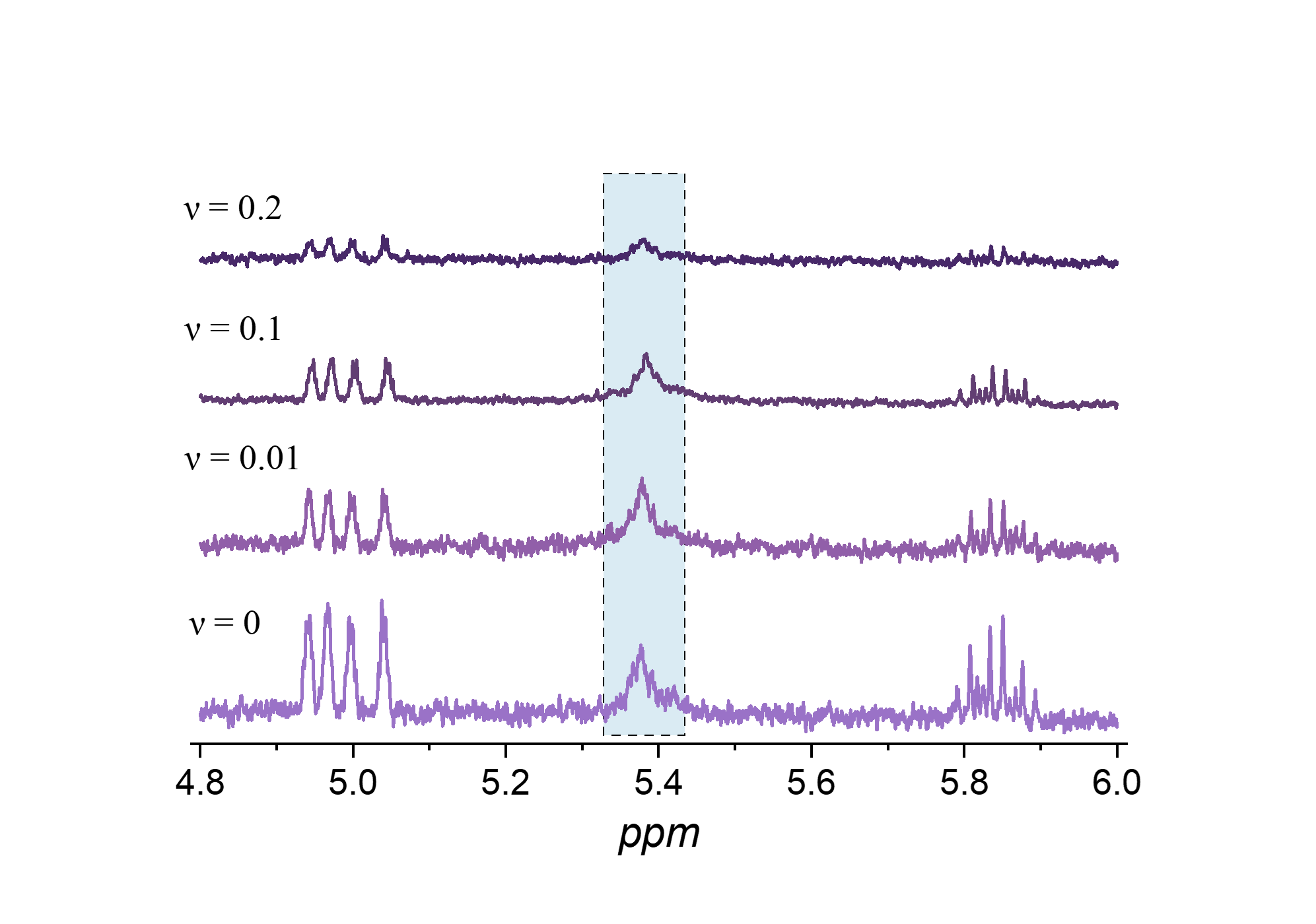


**Fig. S11****.** ^1^H nuclear magnetic resonance spectra of control and target FAPbI_3_ QDs.


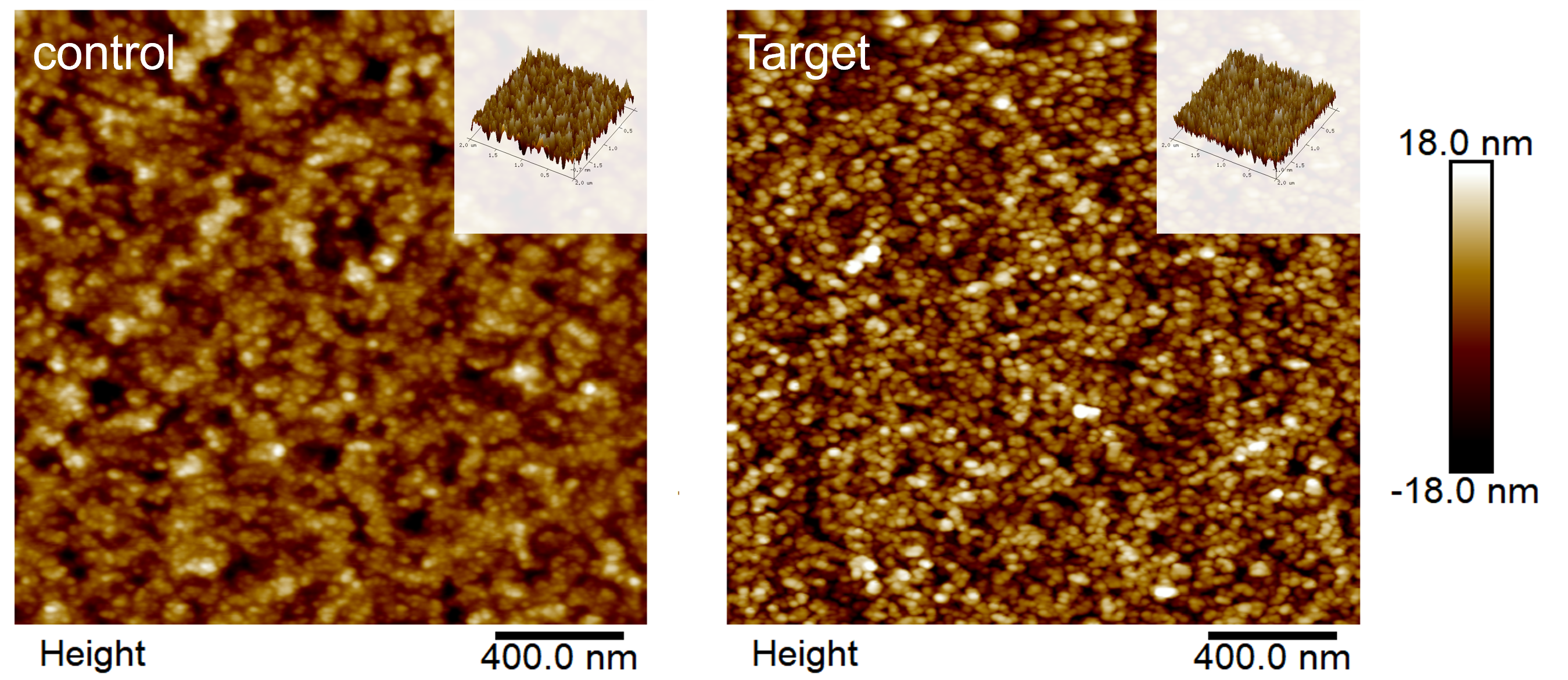


**Fig. S12.** AFM images of control and target QD films.


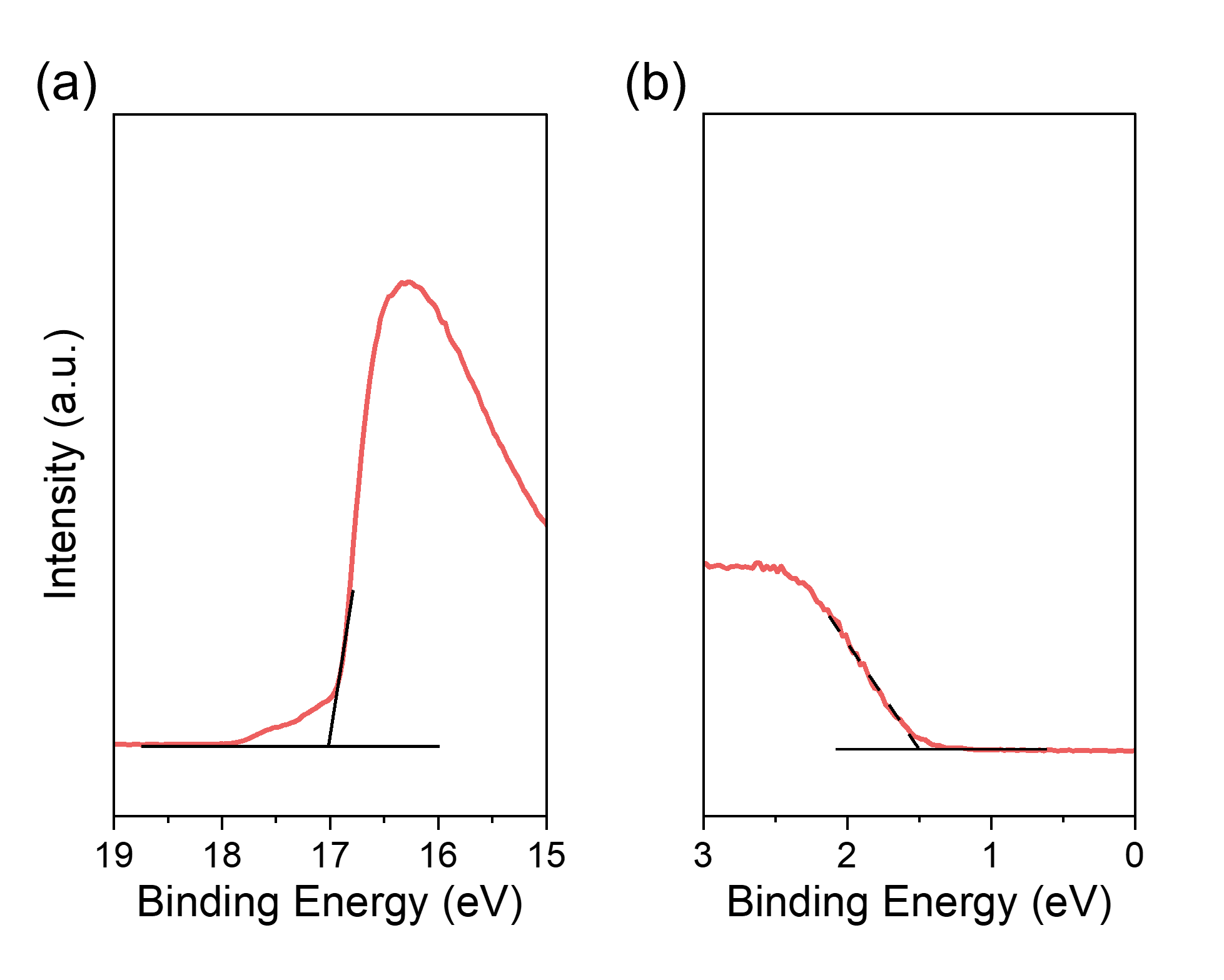


**Fig. S13.** UPS diagram of target FAPbI_3_ QD films.

**
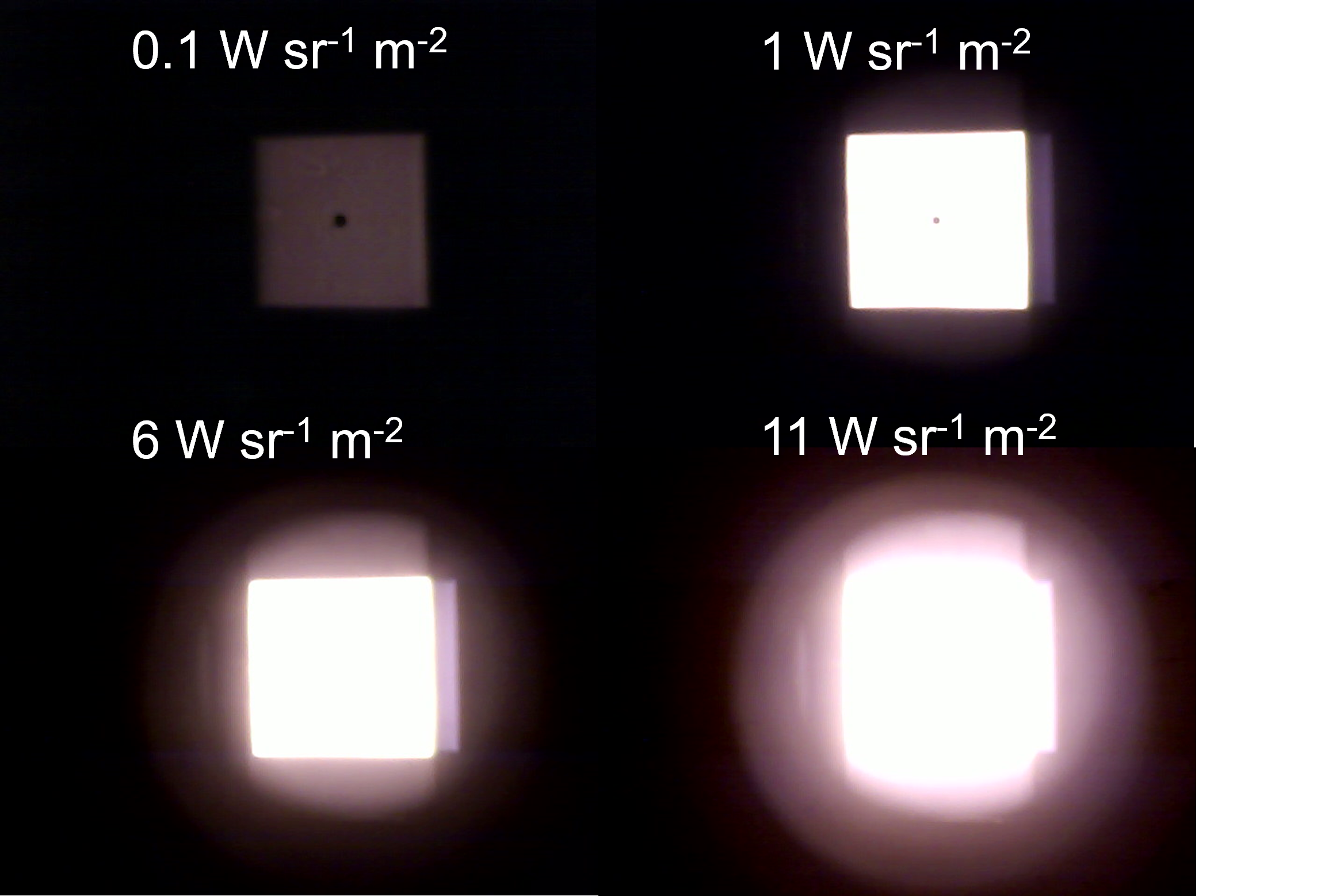
**

**Fig. S14.** Photos of LEDs at various radiance from the infrared camera.


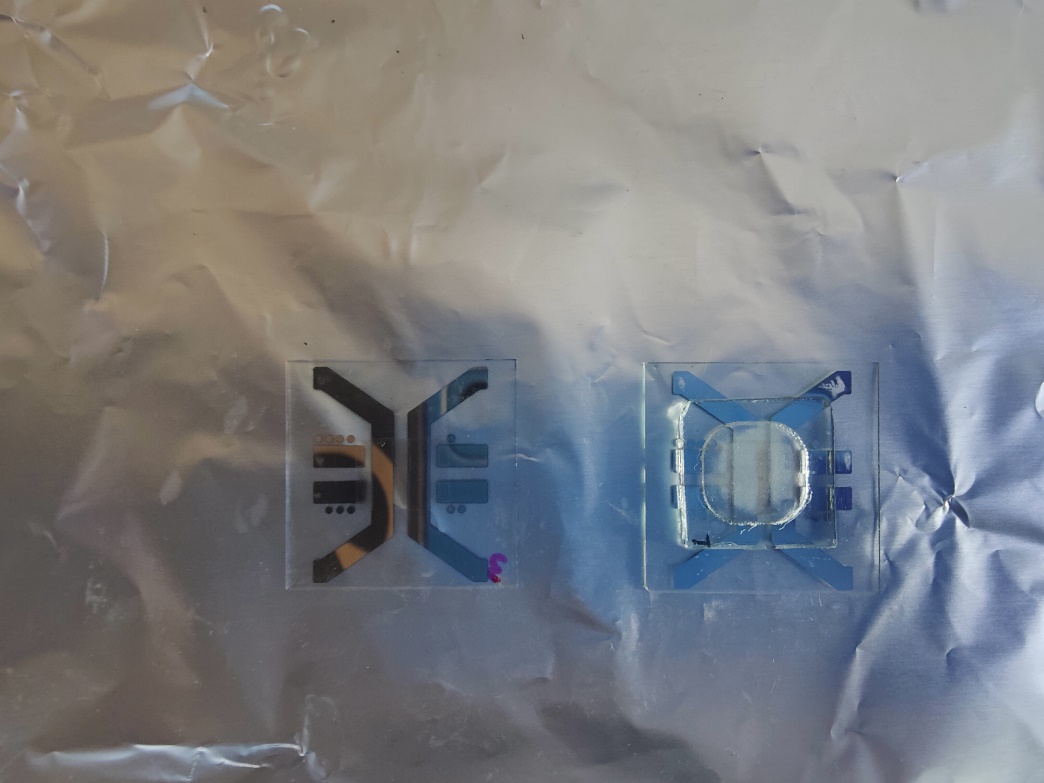


**Fig. S15.** The photos of LEDs before and after encapsulating.
